# Effects of precision feeding and functional amino acid supplementation on *Salmonella*-challenged growing pigs under poor housing conditions

**DOI:** 10.1093/jas/skaf405

**Published:** 2025-12-04

**Authors:** Pedro Righetti Arnaut, Graziela Alves da Cunha Valini, Ismael França, Manoela Trevisan Ortiz, Marllon José Karpeggiane de Oliveira, Danilo Alves Marçal, Antonio Diego Brandão Melo, Alícia Zem Fraga, Amanda Faria de Oliveira, Henrique Gastmann Brand, John Kyaw Htoo, Candido Pomar, Aline Remus, Luciano Hauschild

**Affiliations:** School of Agricultural and Veterinary Sciences, São Paulo State University (UNESP), Jaboticabal, São Paulo 14884-900, Brazil; School of Agricultural and Veterinary Sciences, São Paulo State University (UNESP), Jaboticabal, São Paulo 14884-900, Brazil; School of Agricultural and Veterinary Sciences, São Paulo State University (UNESP), Jaboticabal, São Paulo 14884-900, Brazil; School of Agricultural and Veterinary Sciences, São Paulo State University (UNESP), Jaboticabal, São Paulo 14884-900, Brazil; School of Agricultural and Veterinary Sciences, São Paulo State University (UNESP), Jaboticabal, São Paulo 14884-900, Brazil; School of Agricultural and Veterinary Sciences, São Paulo State University (UNESP), Jaboticabal, São Paulo 14884-900, Brazil; School of Agricultural and Veterinary Sciences, São Paulo State University (UNESP), Jaboticabal, São Paulo 14884-900, Brazil; School of Agricultural and Veterinary Sciences, São Paulo State University (UNESP), Jaboticabal, São Paulo 14884-900, Brazil; School of Agricultural and Veterinary Sciences, São Paulo State University (UNESP), Jaboticabal, São Paulo 14884-900, Brazil; Evonik Brasil Ltda, São Paulo 04711-904, Brazil; Evonik Operations GmbH, Hanau 63457, Germany; Sherbrooke Research and Development Centre, Agriculture and Agri-Food Canada, Sherbrooke, Quebec J1M 0C8, Canada; Sherbrooke Research and Development Centre, Agriculture and Agri-Food Canada, Sherbrooke, Quebec J1M 0C8, Canada; School of Agricultural and Veterinary Sciences, São Paulo State University (UNESP), Jaboticabal, São Paulo 14884-900, Brazil

**Keywords:** amino acids, body composition, immune challenge, individual feeding, nutrient efficiency

## Abstract

Immune system activation impairs pigs’ growth and increases population variability. Individual precision feeding (IPF) takes into account the variability between pigs and improves the efficiency of nutrient utilization. Additionally, functional amino acid (FAA) supplementation reduces the negative impacts of immune system activation. However, the combined effect of IPF and FAA supplementation for immune-challenged pigs remains to be evaluated. We hypothesized that the combined effect of IPF and FAA supplementation would increase nutrient efficiency and reduce the effects of immune activation on pig performance. Therefore, the objective of this study was to evaluate the use of IPF and FAA supplementation on growth performance, body composition, nutrient efficiency, and the blood parameters in growing pigs raised under different sanitary conditions (SC). A total of 120 female pigs were used across two experiments. The pigs were assigned to a good (GSC) or poor (PSC) SC barns, each with 60 pigs. The GSC barn followed strict biosecurity protocols. In the PSC barn, pigs were orally inoculated with *Salmonella* Typhimurium and manure from a commercial swine farm was spread on the pen floor. In each SC barn, pigs were assigned to treatments arranged in a 2 × 2 factorial design. The treatments consisted of two feeding systems (FS): group-phase feeding (GPF) and IPF, and two diets: control (CN) with 100% or supplemented (AA+) with 120% of the SID Met + Cys, Thr, and Trp to Lys ratios above Inraporc recommendations. All pigs had ad libitum access to water and feed during the 28-d experimental period. The PSC increased rectal temperature and haptoglobin concentration compared with d 0 (*P *< 0.05). The IPF impaired the growth performance and body composition under GSC and PSC (*P *< 0.05). The performance of IPF pigs increased when fed AA+ diets under GSC, but no dietary effects were observed under PSC (*P *> 0.05). Group-phase feeding pigs ingested more SID Lys under both SC but had the same Lys efficiency of IPF under PSC (*P *> 0.05). Large fluctuations in body weight and daily feed intake might have impaired the IPF model’s ability to estimate SID Lys requirements. In conclusion, FAA supplementation above the InraPorc recommendations did not mitigate the adverse effects of immune challenge on growth performance or improve nutrient utilization efficiency in IPF pigs.

## Teaser

Precision feeding and functional amino acid supplementation did not increase nutrient efficiency or mitigate the negative effects of immune system activation in growing pigs. The new knowledge acquired in this study improves our ability to precisely feed immune-challenged pigs and highlights the need to consider nutrient utilization by the immune system in the pigs’ requirements.

## Introduction

In swine farms, pigs are constantly exposed to pathogens and unsanitary conditions that activate their immune systems. The release of pro-inflammatory cytokines changes the metabolism and redistributes nutrients away from growth to sustain the immune response (Johnson et al., 1997; Huntley et al., 2018). Consequently, protein and lipid deposition diminishes to increase the amount of nutrients available for the immune system, which, in turn, limits growth performance and nutrient efficiency (Huntley et al., 2018; Valini et al., 2023). The extent to which immune activation impacts growth depends on pathogen load, virulence, and the individual pig’s ability to cope (Wellock et al., 2004; Sandberg et al., 2006, 2007). Therefore, the variability of the nutrient requirement (Ortiz et al., 2024) as well as the demand of some nutrients (Valini et al., 2023) increase during immune system activation.

Conventionally, pigs are fed with group-phase feeding (GPF) programs that provide a single diet for a group of pigs during a phase of the production cycle. These programs estimate the dietary concentration based on the average pig’s requirement (Hauschild et al., 2010). However, nutritional requirements vary between pigs and over time (Pomar et al., 2011; Andretta et al., 2016). These variations are not addressed by GPF, which results in most pigs being overfed and reduces their nutrient efficiency (Hauschild et al., 2010; Pomar et al., 2011; Andretta et al., 2016). When nutrient requirement variability increases, this limitation further impacts the nutrient utilization of immune-challenged pigs.

Emerging tools, such as individual precision feeding (IPF), address this variation by providing tailored diets that match the daily nutritional requirement of each individual animal (Hauschild et al., 2012). Studies have shown that feeding pigs with IPF significantly reduced Lys intake by 27% and nitrogen (N) excretion by 30% without compromising growth (Andretta et al., 2014, 2016). These findings suggest that IPF could be a feeding strategy to improve nutrient efficiency of immune-challenged pigs, since it accounts for individual variability, which becomes more relevant under such conditions. Although promising, the effectiveness of IPF in immune-challenged pigs remains to be fully evaluated.

Additionally, the metabolic changes resulting from immune activation increase the need for specific amino acids (Reeds et al., 1994; Le Floc’h et al., 2018). These amino acids are often referred to as “functional” AA (FAA) due to their role in the synthesis of immune components. For instance, the supplementation of the FAA Met, Thr, and Trp was shown to mitigate the impact of immune stimulation (Van der Meer et al., 2016; Rodrigues et al., 2021; Valini et al., 2023). These FAA improve antioxidant capacity, prevent pathogen translocation, and minimize the long-lasting effects of the immune response, respectively (Le Floc’h et al., 2018; Rodrigues et al., 2021). Therefore, the association of IPF and FAA could increase the performance of immune-challenged pigs and increase their nutrient efficiency.

We hypothesized that the combined effect of IPF and FAA would reduce the effects of immune system activation on pig performance and increase nutrient efficiency. Thus, the objective of this study was to evaluate growth performance, body composition, Lys and N efficiency, and blood parameters of sanitary-challenged pigs fed with different feeding systems (**FS**), with or without FAA supplementation.

## Material and Methods

All experimental procedures applied in this trial followed the Brazilian National Council of the Control of Animal Experimentation (CONCEA) and were reviewed and approved [protocol No. 0607/22] by the Ethical Committee on Animal Use (CEUA) of the School of Agricultural and Veterinary Sciences of São Paulo State University (UNESP).

### Animal husbandry and experimental design

This study was conducted simultaneously under two different sanitary conditions (SC) at the Swine Research Facility of São Paulo State University, Brazil. One hundred and twenty female pigs (Pietran × [Large White × Landrace]) with an initial BW of 16.1 ± 2.1 kg were divided and housed in two similar growing-finishing barns with 60 pigs each. Each facility consisted of a single pen with concrete floors and controlled temperature and humidity through an automated evaporative pad cooling system (Big Dutchman, Araraquara, SP, Brazil) and exhaust fans. The room temperature was maintained between 18 and 24°C and relative humidity between 60 and 70%. The pen was equipped with 8 nipple drinkers and 5 automated feeding stations (Automatic Intelligent Precision Feeders; University of Lleida, Spain; Pomar et al., 2011). The feeders can identify the pigs through electronic ear tags with individual identification numbers, allowing the pigs to be housed in the same pen and receive the prescribed dietary treatment. Additionally, the feeders can mix up to 4 different diets according to proportions that are registered in their software and record individual feed intake. The feeders were calibrated and tested every day during the adaptation and experimental periods. A 14-d period was used to adapt the pigs to the feeders and collect individual baseline information on daily feed intake (DFI), BW, and daily gain (DG). After the adaptation period, the barns were used to create either a poor (PSC) or good SC (GSC). Under GSC and PSC, the pigs (23.15 ± 2.81 and 22.82 ± 2.78 kg BW, respectively) were distributed as complete randomized block design with a 2 × 2 nested factorial arrangements. The treatments were randomly assigned within each weigh block and consisted of two feeding systems: GPF and IPF; and two diets: control (CN) or supplemented (AA+) with Met + Cys, Thr, and Trp at 120% of InraPorc (Van Milgen et al., 2008) recommendations.

Before the trials began, rectal swabs performed individually on all pigs to screen for the presence of *Salmonella* species, and all pigs were negative. A 12-h period of light was set from 0700 h to 1900 h and the room temperature was gradually decreased from 22°C at the beginning to 18 °C at the end of the experimental period, which lasted 28 d. During the adaptation and experimental periods, the pigs had ad libitum access to water and feed.

### Sanitary conditions

The barns were used to create two different SC. All pigs in the PSC barn were induced to an enteric infection on d 0 through oral inoculation of 5 mL solution of brain heart infusion broth (CM 1135, Oxoid, Thermo Fisher Scientific, United Kingdom) containing a total of 2 × 10^9^ CFU of *Salmonella* Typhimurium. Additionally, fresh manure from a commercial pig farm was evenly spread on the pen floor (1.7 kg/m^2^; Valini et al., 2023). The strain *Salmonella enterica* subsp. enterica serovar Typhimurium (RL0971/09) was originally isolated from swine feces and it was naturally resistant to nalidixic acid (Nal+; 25 µg/mL). The inoculum was prepared according to Wood et al. (1991) and Oliveira et al. (2010) at the Laboratory of Ornitopathology (Department of Veterinary Pathology, FCAV/UNESP, Jaboticabal-SP, Brazil) 72 h before inoculation at 37°C in buffered peptone water and diluted with sterile phosphate buffer saline. The solution was inoculated by oral gavage after 6 h of fasting and no water consumption for 1 h. The PSC barn was not cleaned during the experimental period. In contrast, the GSC barn was cleaned twice a day with pressurized water. Disinfected clothing and footwear were required before entering the barn. In the GSC barn, pigs received an oral inoculation of 5 mL of brain heart infusion broth without *Salmonella* Typhimurium on d 0 to mimic the same handling procedure applied to PSC pigs. Rectal swabs were performed weekly on all GSC pigs to screen for the presence of the *Salmonella* Typhimurium strain inoculated under PSC to monitor for possible cross-contamination. No pigs in either condition received any medications or preventive treatments before or during the trial.

### Feeding systems and diets

The pigs were randomly assigned to GPF or IPF feeding systems on d 0 in both SC. Group-phase pigs were fed with a diet containing 1.11% SID Lys for the duration of the trial. The SID Lys concentration of GPF pigs corresponded to the SID Lys requirement of the animal placed in the 80th percentile pig. The 80th percentile pig was chosen assuming that its SID Lys requirement represented the dietary concentration that maximizes population growth performance (Hauschild et al., 2010; Remus et al., 2020). To determine the 80th percentile for this trial, we used the average of the 80th percentile of all pigs during the 3 d before the beginning of the trial. Pigs in both GSC and PSC barns assigned to GPF treatment received the same SID Lys concentration. In contrast, pigs assigned to the IPF group received tailored diets adjusted to their daily SID Lys requirement according to the model of Hauschild et al. (2012). The model uses an empirical and a mechanistic component to estimate the SID Lys requirements of each individual. The empirical component forecast the DFI, BW, and DG based on each pig’s feed intake and growth patterns. The mechanistic component uses the forecasted DFI, BW, and DG as input in factorial equations to estimate the SID Lys requirement for maintenance and growth (Hauschild et al., 2012). Lysine requirements for maintenance were estimated adding basal endogenous losses [0.313 g Lys/kg dry matter (DM) × DFI] to the losses related to desquamation in the digestive tract (0.0045 g Lys/kg BW^0.75^ × BW^0.75^), and losses related to body protein turn over (0.0239 g Lys/kg BW^0.75^ × BW^0.75^; Van Milgen et al., 2008). The SID Lys requirements for growth were estimated assuming that 16% of the DG is protein (De Lange et al., 2003) and that 7% of the protein deposition is composed of Lys (Mahan and Shields, 1998), and that the efficiency of Lys retention from dietary digestible Lys is 72% (Möhn et al., 2000). Individual BW was recorded twice a week and DFI measured daily by the feeding stations to be used as input in the IPF model.

Four experimental diets were formulated for this trial: A1, B1, A2, and B2 (Table 1). Diets A (high nutrient density) were formulated to meet the requirement of the most demanding pig at the beginning of the growing phase (1.30% SID Lys). Meanwhile, diets B (low nutrient density) were formulated to meet the requirements of the least demanding pig at the end of the growing phase (0.47% SID Lys) based on IPF model estimation using previous data (Valini et al., 2023). Diets A1 and B1 were formulated with 100% (CN) and diets A2 and B2 with 120% (AA+) of the SID Met + Cys, Thr, and Trp: Lys. Therefore, the SID Lys requirements of the pigs in the GPF and IPF treatments were achieved with either a blend of CN (A1 and B1) or AA+ (A2 and B2). Amino acids were balanced in the CN diets in fixed proportions relative to SID Lys, with ratios of 30% Methionine, 60% Methionine + Cysteine, 65% Threonine, 20% Tryptophan, 60% Isoleucine, 100% Leucine, 70% Valine, 50% Phenylalanine, 32% Histidine, and 42% Arginine (Van Milgen et al., 2008). Minerals (Ca, P, and Na) and energy content were calculated using the NRC (2012) recommendations for the phase from 25 to 50 kg BW. The diets were steam-pelleted (2.5 mm) and no in-feed antibiotics were added.

**Table 1.** Ingredients and chemical composition of experimental diets A1, A2, B1, and B2 (as-fed basis)^1^

Item, %	A1	A2	B1	B2
***Feedstuff***				
** Corn**	67.7	67.1	90.8	92.1
** Soybean meal**	26.0	26.1	–	–
** Soybean oil**	1.27	1.33	0.54	0.05
** Kaolin (inert)**	–	–	4.85	3.97
** Dicalcium phosphate**	1.41	1.41	1.22	1.21
** Limestone**	0.77	0.77	0.72	0.73
** Salt**	0.22	0.22	0.23	0.23
** L-lysine^2^**	0.86	0.86	0.49	0.49
** DL-methionine^2^**	0.29	0.45	0.01	0.06
** L-threonine^2^**	0.29	0.46	0.09	0.15
** L-tryptophan^2^**	0.09	0.14	0.05	0.07
** L-valine**	0.18	0.18	0.03	0.02
** L-isoleucine**	0.12	0.12	0.05	0.05
** Maltodextrin**	0.50	0.50	0.50	0.50
** Min/vit premix^3^**	0.12	0.12	0.12	0.12
** Antifungic**	0.10	0.10	0.10	0.10
** Choline chloride**	0.06	0.06	0.06	0.06
** Antioxidant**	0.01	0.01	0.01	0.01
** Mycotoxin adsorbent**	0.10	0.10	0.10	0.10
***Calculated chemical composition***				
** Dry matter, %**	83.54	82.95	84.65	84.40
** Crude protein, %**	18.36	18.61	7.96	8.15
** Metabolizable energy, kcal/kg**	3,360	3,370	3,150	3,170
** Net energy, kcal/kg **	2,480	2,480	2,480	2,480
** Total Calcium**	0.74	0.74	0.59	0.59
** Total Phosphorus**	0.55	0.55	0.41	0.41
** STTD Phosphorus**	0.34	0.34	0.27	0.27
***Estimated SID amino acid^4^***				
** Lysine**	1.31	1.28	0.50	0.51
** Methionine + Cysteine**	0.77	0.91	0.30	0.36
** Threonine**	0.84	1.02	0.34	0.41
** Tryptophan^5^**	0.26	0.31	0.09	0.11
** Methionine**	0.51	0.66	0.15	0.19
** Cysteine**	0.24	0.25	0.16	0.15
** Valine**	0.86	0.85	0.40	0.37
** Arginine**	0.94	0.95	0.38	0.35
** Isoleucine**	0.71	0.71	0.32	0.30
** Leucine**	1.29	1.32	0.90	0.85
** Histidine**	0.41	0.41	0.22	0.21
** Phenylalanine**	0.71	0.72	0.37	0.35

1 Diets A and B were formulated with 1.30 and 0.47% SID Lys, respectively. Diets 1 and 2 were formulated with 100% and 120%, respectively, of the Met + Cys, Thr, and Trp: Lys recommended by InraPorc (Van Milgen et al., 2008).

2 Amino acids BioLys, MetAMINO, ThreAMINO, and TrypAMINO were provided by Evonik Nutrition & Care GmbH (Hanau-Wolfgang, Germany). Supplied per kg of product (as-fed basis): 62.4, 99.0, 98.5, and 98.0% of L-lysine, DL-methionine, L-threonine, and L-tryptophan, respectively.

3 Mineral and vitamin premix provided by Nutri—Guabi Nutrição e Saúde Animal (Sales Oliveira-São Paulo, Brazil). Supplied per kg of diet (as-fed basis): Manganese, 40 mg; Copper, 15 mg; Iron, 24.93 mg; Cobalt, 0.168 mg; Iodine, 1.416 mg; Zinc, 74.971 mg; Folic acid, 0.32 mg; D-Pantothenic acid, 14.8 mg; Biotin, 0.04 mg; Niacin, 28 mg; Selenium, 0.25 mg; Vitamin A, 6000 IU; Vitamin B_1_, 1.2 mg; Vitamin B_12_, 22 mcg; Vitamin B_2_, 4.4 mg; Vitamin B_6_, 1.4 mg; Vitamin D_3_, 1400 IU; Vitamin E, 26 IU; Vitamin K_3_, 2.16 mg.

4 Estimated applying the digestibility coefficients obtained with EvaPig on total amino acid concentrations. SID = standardized ileal digestible, STTD = standardized total tract digestible.

5 Estimated values used to formulate the diets.

Dietary total AA and CP concentrations were analyzed using the Association of Official Analytic Chemists method (AOAC, method 994.12, AOAC, 2010) at the AMINOLab of Evonik (Germany). The estimated SID AA composition was estimated by applying the digestibility coefficients obtained with EvaPig (version 2.0.3, METEX NØØVISTAGO, INRAE and AFZ, France) to the analyzed total AA concentration (Table 1).

### Data collection

All animals were used for growth performance and fecal score evaluation and fecal ST quantification (*n* = 15 pigs/treatment). Additionally, a group of 40 pigs (*n* = 10 pigs/treatment) with the closest BW to the average of its experimental group were selected for the evaluation of rectal temperature, blood sampling, body composition, and nutrient efficiency analyses. All procedures were conducted in both SC on the same experimental day, and the same animals were used for the same measures over growth.

#### Growth performance

The BW and DFI of all pigs were measured twice a week and daily, respectively, starting two weeks before the trial. These data were used to estimate the 80th percentile pig SID Lys requirement at the beginning of the trial, thus stablishing the GPF requirement for the phase, and to calculate SID Lys requirements in the IPF throughout the experiment. For the growth performance analysis, the BW and feed intake were used to assess the ADG, ADFI, and G:F.

#### Rectal temperature and fecal Salmonella shedding

Rectal temperature was measured daily using digital thermometers (Accumed-Glicomed, Rio de Janeiro, Brazil) from d 0 to d 7. On d 0, the temperature was measured before introducing the pigs to the SC and dietary treatments.

Fecal samples were collected from all PSC pigs on d 3, 7, 14, 21, and 28 for the detection of *Salmonella*. The samples were individually collected with rectal stimulation. Fresh fecal samples were serially diluted in phosphate buffer saline (1:10) until they reached the final concentration of 10^−6^. One hundred microliters from each dilution were plated on brilliant green agar containing Nal+. Plates were incubated at 37°C for 24 h. In the absence of growth, an equal volume of Rappaport broth (CM0669, Oxoid, United Kingdom), prepared in double concentration, was added to the falcon tubes containing the homogenized sample in PBS (1:10). The sample tubes were incubated at 37°C for 24 h and plated again in Nal+ at 37°C for 24 h. The number of CFU per gram of feces was transformed into Log10 for further analysis. Plates with at least one colony of *Salmonella* Typhimurium were considered positive and were used to determine the percentage of positive pigs in each treatment. Meanwhile, the rectal swabs collected before and during the trial to screen for the presence of *Salmonella* Typhimurium were serially diluted in (1:10) until they reached the final concentration of 10^−6^. From each dilution, 0.1 mL was plated on brilliant green agar (CM0263, Oxoid, United Kingdom) and incubated at 37°C for 24 h.

#### Blood sampling and analysis

Blood samples were collected from the jugular vein on d 0, 7, and 28, after a minimum of 6 h fasting. On d 0, the samples were collected before introducing the pigs to the SC, feeding systems, and diets. In each sampling, serum tubes (BD, New Jersey, USA) were collected for haptoglobin, albumin, total protein, urea, creatinine, IgA, IgG, and triglyceride analysis. The tubes were allowed to clot for 2 h at room temperature before centrifugation for 10 min at 3,000×*g* at 4°C and stored at −80°C. The haptoglobin and albumin serum concentrations were determined with sodium dodecyl sulfate-polyacrylamide gel electrophoresis (Weber and Osborn, 1969). The molecular weight and protein fraction concentrations were determined by computer densitometry (Shimadzu 9301, Kyoto, Japan) with a simple scanner. The reference curves for densitometry evaluation of protein bands were constructed as linear standard curves based on the readings of the standard marker. The serum haptoglobin, albumin, IgA, and IgG concentration were then corrected using the serum total protein analysis. The serum concentrations of total protein, urea, creatinine, and triglycerides were determined by the biuret method (Labtest, Lagoa Santa, Brazil).

#### Body composition analysis

Body composition was estimated at the beginning and the end of the experimental period. The pigs were anesthetized after 6 h of fasting by intramuscular injection of xylazine (1.5 mg/kg BW) and ketamine (15 mg/kg BW) and scanned in prone position with dual-energy X-ray absorptiometry equipment ([DXA], GE Lunar Prodigy Advance; GE Healthcare, USA). The estimated lean and fat masses were converted into their protein and lipid chemical equivalents according to Pomar and Rivest (1996). These data were used to determine the body protein and lipid masses. Daily protein deposition (PD) and lipid deposition (LD) were calculated as the difference between the respective body constituents estimated by the DXA at the beginning and end of the experimental period.

#### Lysine and nitrogen utilization efficiencies

The SID Lysine (_k_Lys) and N utilization efficiencies were obtained using only the pigs scanned with the DXA. Nitrogen and SID Lys intake were calculated by multiplying the individual ADFI by its respective concentration in the blend of feeds provided. Nitrogen intake and retention were estimated assuming that dietary CP and PD contains 6.25% of N. Values of N efficiency were expressed as the percentage of the intake that was retained. Lysine efficiency was calculated by dividing the amount of available and retained Lys. Available Lys was estimated subtracting the maintenance from the intake. Lysine maintenance requirements were calculated using the maintenance formulas to estimate the individual requirements. Lysine retention values were estimated assuming that 7% of the PD is Lys (Mahan and Shields, 1998).

### Statistical analysis

Since there was no repetition of the application of the SC factor, the statistical analysis was conducted separately for each SC. Data were tested for normality using the UNIVARIATE procedure of SAS (version 9.4, SAS Institute Inc., North Carolina, USA) and analyzed using the MIXED procedure as a randomized complete block design. Feeding systems, diets, and their interactions were included as fixed effects while the blocks of BW were included as a random effect in the statistical model. The Wilcoxon Rank Sum test were used to analyze the frequency and excretion of *Salmonella* Typhimurium, respectively. Blood parameters, rectal temperature, BW, body protein, body lipid, ADG, ADFI, and SID Lys intake were evaluated as repeated measures over time using the co-variance matrix with the lowest AIC value. Different periods of time were used in the repeated measures variable analysis. The sampling days were used for blood parameters and rectal temperature. The initial and final experimental days were used as fixed effects for BW, body lipid, and body protein analysis. Finally, a fixed week effect was used in the respective analysis of ADG, ADFI and SID Lys intake. Time effect was further analyzed using a Tukey adjustment. The individual pig was considered the experimental unit. Effects were considered significant when *P *≤ 0.05.

## Results

### General overview

A clinical visual examination indicated that most pigs under PSC experienced nausea, diarrhea, and lethargy during the first week. During the experimental period, three pigs from the PSC barn were removed from the experiment owing to lameness and arthritis. The decision to remove these pigs was based on the observation of clinical signs, monitoring of body temperature and a sustained reduction in feed intake. The pigs were medicated following veterinarian guidelines and removed from the statistical analysis. All swabs performed in the GSC pigs were negative for the *Salmonella* Typhimurium strain inoculated under the PSC (data not shown), indicating the absence of cross-contamination between SC.

**Figure 1.**
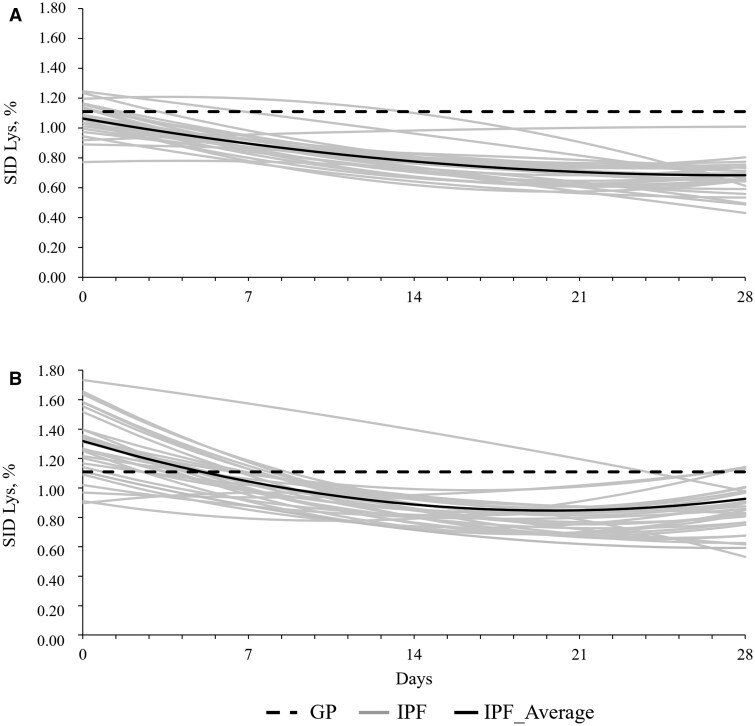
Estimated dietary SID Lys concentration requirements in group-phase (GPF) or individual precision feeding (IPF) systems for pigs raised in good (A) or poor (B) sanitary conditions. GPF pigs received a 1.11% SID Lys concentration diet over the entire trial in both sanitary conditions. IPF pigs were individually fed with daily tailored diets to meet their individual requirements in both sanitary conditions. The good sanitary condition was achieved with biosecurity protocols whereas in the poor condition, pigs were orally inoculated with *Salmonella* Typhimurium and manure from a commercial swine farm was spread on the pen floor.

Descriptive analysis showed potential differences across SC in dietary SID Lys requirement patterns for IPF pigs (Figure 1). The coefficient of variation (CV) of individual SID Lys requirements in IPF pigs was lower under GSC (19%) than under PSC (24%). The sanitary challenge imposed on PSC pigs reduced their ADFI in both feeding systems when compared with the week before the trial (1,027 vs. 637 g/d). As a result, the average SID Lys concentration estimated for PSC pigs in the first week increased when compared with GSC pigs (1.17% vs. 0.98% SID Lys, respectively). However, even with the increased SID Lys concentration, GPF and IPF pigs in the GSC ingested, respectively, 35% and 26% more SID Lys than PSC pigs in the same feeding system. In the GSC, pigs receiving CN diets within IPF, ingested 29% less Lys, 29% less Thr, 30% less Trp and 34% less Met than GPF pigs. In the GSC, pigs receiving AA+ diets within IPF, ingested 33% less Lys, 32% less Thr, 33% less Trp and 38% less Met than GPF pigs. The Thr: Lys was 0.64 for GPF and 0.65 for IPF, Met: Lys was 0.39 for GPF and 0.36 for IPF, and Trp: Lys was 0.20 for both FS receiving CN diets within GSC. For AA+ diets within GSC, Thr: Lys was 0.80 for both FS, Met: Lys was 0.53 for GPF and 0.50 for IPF, and Trp: Lys was 0.24 for IPF and 0.25 for GPF.

Descriptive analysis showed that, during the 28-d trial, GPF pigs fed with AA+ diets in the PSC consumed 7% less SID Lys and 12%, 14%, and 13% more SID Met + Cys, Thr, and Trp than CN pigs, respectively. However, the ADFI of GPF pigs fed AA+ diets was 99 g lower than that of CN pigs during the first experimental week (709 vs. 610 g/d, respectively). Consequently, in the first experimental week, GPF pigs fed with AA+ diets ingested 15% less SID Lys and only 2, 5, and 3% more SID Met + Cys, Thr, and Trp than CN pigs, respectively. On the other hand, in the overall 28-d trial, IPF pigs fed with AA+ diets under PSC consumed 7, 30, 33, and 32% more SID Lys, Met + Cys, Thr, and Trp when compared with CN pigs, respectively. Similar results were observed in the first experimental week with IPF pigs fed with AA+ diets ingesting 4, 26, 29, and 27% more SID Lys, Met + Cys, Thr, and Trp than CN pigs, respectively.

Overall, in the 28 experimental days, in the PSC, pigs receiving CN diets within IPF, ingested 33% less Lys, 32% less Thr, 33% less Trp and 38% less Met than GPF pigs. In the PSC, pigs receiving AA+ diets within IPF, ingested 33% less Lys, 32% less Thr, 35% less Trp and 43% less Met than GPF pigs. The Thr: Lys was 0.64 for GPF and 0.65 for IPF, Met: Lys was 0.39 for GPF and 0.36 for IPF, and Trp: Lys was 0.20 for both FS receiving CN within PSC. In the AA+ diets, the Thr: Lys was 0.80 and Trp: Lys was 0.24 for both FS, whereas Met: Lys was 0.50 for GPF and 0.47 for IPF within PSC.

### Rectal temperature and *Salmonella* Typhimurium shedding

**Figure 2.**
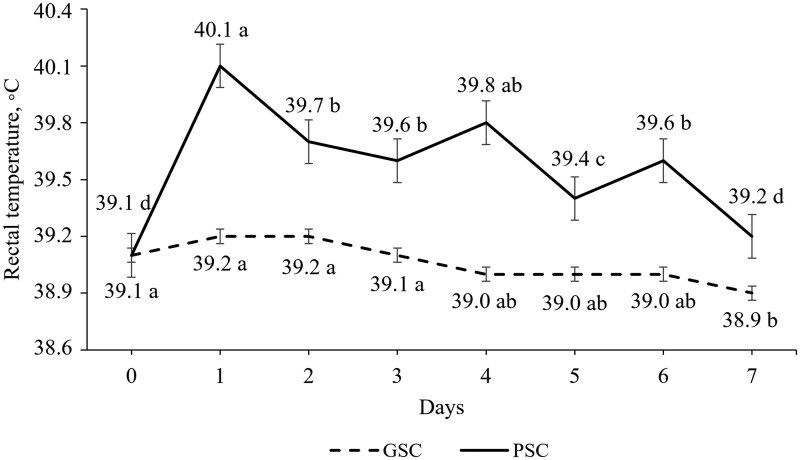
Rectal temperature of pigs assigned to good (GSC) or poor (PSC) sanitary conditions during the first week of trial. The good sanitary condition was achieved with biosecurity protocols whereas in the poor condition, pigs were orally inoculated with *Salmonella* Typhimurium and manure from a commercial swine farm was spread on the pen floor. Different letters indicate differences between days in each sanitary condition (*P *< 0.05). No significant effects of feeding systems, diets, and their interactions were observed (*P *> 0.05).

**Figure 3.**
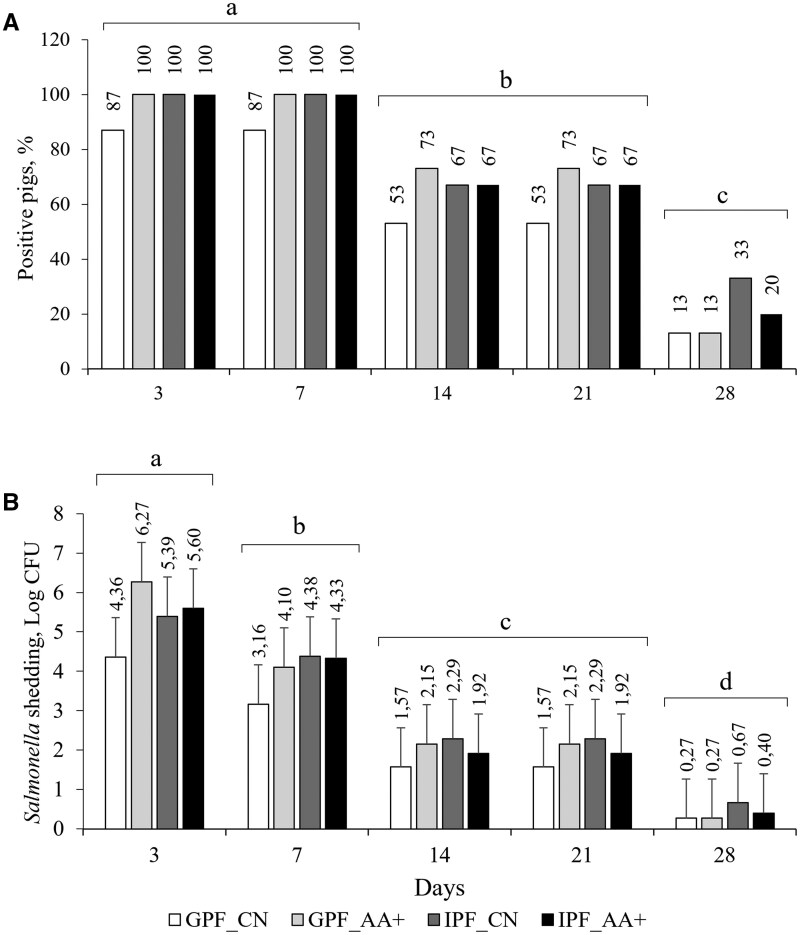
Percentage of pigs positive for *Salmonella* Typhimurium shedding (A) and the Log of colony forming units (CFU) of *Salmonella* Typhimurium shedding (B) at 5 sampling days of poor sanitary condition pigs. The poor condition was achieved with oral inoculation of *Salmonella* Typhimurium and manure from a commercial swine farm was spread on the pen floor. Pigs were fed with either a control (CN) or functional amino acids supplemented (AA+) diet in group-phase (GPF) or individual precision (IPF) feeding systems. Different letters indicate differences between days (*P *< 0.05). No significant effects of feeding systems, diets, or their interactions were observed (*P *> 0.05).

No differences (*P *> 0.05) in rectal temperature, percentage of *Salmonella* positive pigs, and *Salmonella* Typhimurium shedding (Figure 3) were observed for the main effects of FS, diet, and their interactions (Figure 2). The GSC pigs’ rectal temperature remained stable during the first week of the trial, declining on d 7 (*P *< 0.05). Conversely, PSC pigs’ rectal temperature sharply increased on d 1 after the inoculation with a graded reduction until d 7 (*P *< 0.05).

In the PSC barn, all pigs were at least 1 d positive for *Salmonella* Typhimurium shedding (Figure 3). The greatest percentages of positive pigs were observed on d 3 and d 7 (*P *< 0.05) with a significant decrease on d 14, 21, and 28 (*P *< 0.05). Similarly, the *Salmonella* shedding decreased linearly towards the end of the experimental period (*P *< 0.05).

### Animal performance and body composition

#### Good sanitary conditions

**Figure 4.**
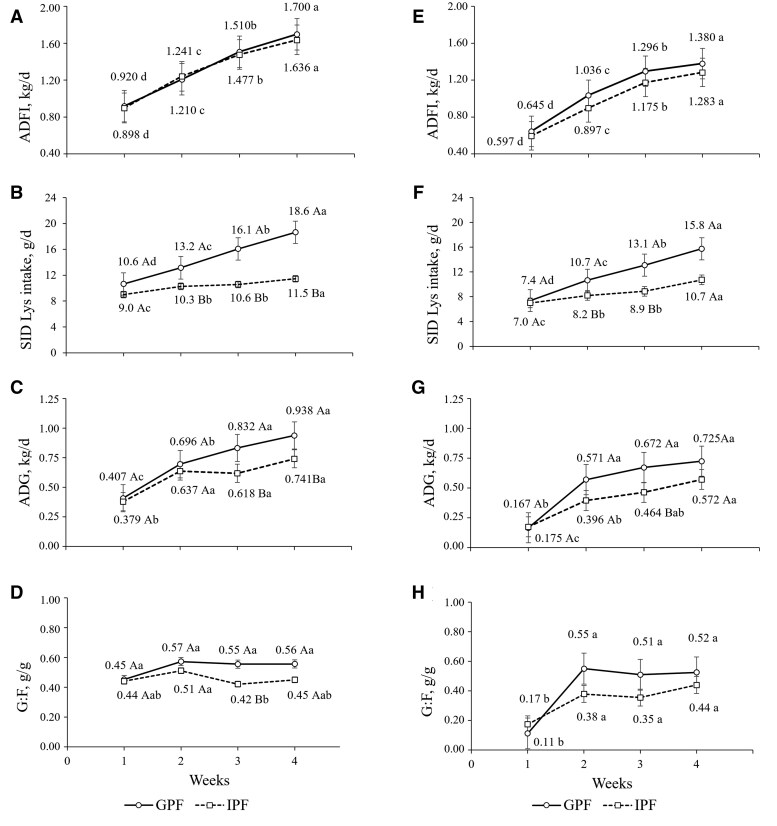
Average daily feed intake (ADFI) SID Lys intake, average daily gain (ADG), and feed efficiency (G:F) of pigs fed with group-phase (GPF) or individual precision feeding (IPF) systems and raised in good (A–D) or poor (E–H) sanitary conditions. The good sanitary condition was achieved with biosecurity protocols whereas in the poor condition, the pigs were orally inoculated with *Salmonella* Typhimurium and manure from a commercial swine farm was spread on the pen floor. ^Aa–Cc^Uppercase indicate differences (*P *< 0.05) between feeding systems within each week and lowercase letters indicate differences (*P *< 0.05) over time (weeks) within feeding system by multiple comparisons using a Tukey adjustment. Values are least square means.

The growth performance and body composition of pigs raised under GSC or PSC are shown in Table 2. Interactions between feeding systems and time were observed for ADG and SID Lys intake under GSC and PSC (*P *< 0.05; Figure 4). Under GSC, a triple interaction between feeding systems, diet, and time demonstrated that although IPF pigs had lower BW, it increased on d 28 when they were fed with AA+ diets (*P *< 0.05). Similarly, AA+ diets improved the ADG and G:F of IPF pigs (*P *< 0.05) in the same 28-d period. Body protein was greater in GPF pigs (*P *< 0.05), regardless of the dietary treatment. The ADFI was not affected (*P *> 0.05) by the FS or FAA supplementation (Figure 4). Main effects of feeding systems and dietary treatments were observed for PD where group-phase pigs had greater PD than IPF (*P *< 0.05) and AA+ pigs greater PD than pigs fed with CN diets (*P *= 0.05).

**Table 2.** Growth performance and body composition of pigs raised in good or poor sanitary conditions and fed with control (CN) or functional amino acid supplemented (AA+) diets in group-phase (GPF) or individual precision (IPF) feeding systems^1^

	GPF		IPF			*P*-value						
Item	CN	AA+	CN	AA+	SEM	FS	Diet	T	FS × Diet	FS × T	Diet × T	FS × Diet × T
***Good sanitary conditions***												
***Initial conditions***												
** BW, kg**	23.2	23.1	23.2	23.3	0.377	0.49	0.84		0.55			
** Body protein, kg**	2.95	2.94	2.89	3.00	0.055	0.99	0.68		0.59			
** Body lipid, kg**	4.44	4.60	4.51	4.62	0.039	0.62	0.09		0.75			
*** Final conditions***												
** BW, kg**	43.4	43.0	38.8	41.0	0.975	0.07	0.23	0.01	0.20	0.01	0.08	0.05
** Body protein, kg**	6.13	6.31	5.50	5.81	0.186	0.09	0.27	0.01	0.49	0.01	0.16	0.47
** Body lipid, kg**	6.24	5.93	6.30	6.12	0.108	0.34	0.56	0.01	0.94	0.28	0.41	0.65
***Overall p*erformance**												
** ADG, g/d**	722^Aa^	711^Aa^	552^Ab^	636^Aa^	16.37	0.01	0.01	0.01	0.01	0.01	0.75	0.57
** ADFI, g/d**	1303	1326	1257	1337	24.16	0.67	0.12	0.01	0.36	0.20	0.21	0.32
** G: F, g/g**	0.55^Aa^	0.54^Aa^	0.44^Ab^	0.48^Aa^	0.009	0.01	0.47	0.01	0.03	0.03	0.99	0.78
** PD, g/d**	114	120	91	102	3.002	0.01	0.05		0.59			
** LD, g/d**	61	50	63	58	2.672	0.34	0.16		0.57			
***Poor sanitary conditions***												
*** Initial conditions***												
** BW, kg**	23.0	22.8	22.8	23.1	0.368	0.83	0.82		0.19			
** Body protein, kg**	2.98	2.96	2.90	3.02	0.056	0.89	0.68		0.53			
** Body lipid, kg**	4.45	4.42	4.47	4.52	0.034	0.40	0.89		0.57			
*** Final conditions***												
** BW, kg**	38.6	37.2	33.1	35.6	0.751	0.09	0.75	0.01	0.27	0.01	0.57	0.14
** Body protein, kg**	5.56	5.65	4.72	4.80	0.160	0.03	0.73	0.01	0.50	0.01	0.77	0.66
** Body lipid, kg**	5.42	5.58	5.20	5.31	0.067	0.51	0.58	0.01	0.57	0.03	0.28	0.90
***Overall p*erformance**												
** ADG, g/d**	554	515	367	447	19.73	0.01	0.60	0.01	0.11	0.01	0.72	0.88
** ADFI, g/d**	1112	1036	972	1009	24.94	0.09	0.91	0.01	0.25	0.41	0.71	0.84
** G: F, g/g**	0.49	0.49	0.37	0.43	0.022	0.07	0.25	0.01	0.24	0.35	0.57	0.77
** PD, g/d**	92	96	63	66	5.091	0.01	0.70		0.98			
** LD, g/d**	34	40	26	30	1.898	0.02	0.19		0.78			

SEM = standard error of means, BW = body weight, ADG = average daily gain, ADFI = average daily feed intake, G: F = feed efficiency, PD = protein deposition, LD = lipid deposition.

1 Data were analyzed considering feeding systems (FS), diets (Diet), time (T) and their interaction as fixed effects. Time was not included as fixed effect for the initial conditions analysis and PD and LD.

Aa–Bb Different lowercase and uppercase superscripts indicate differences (*P *< 0.05) between FS within Diet and Diets within FS, respectively.

Three pigs were removed from the growth performance analysis in poor sanitary condition due to lameness and arthritis: one pig from GPF_CN and two pigs from IPF_CN treatments. Additionally, two outliers were removed from the analysis in good sanitary condition: two pigs from IPF_AA+ treatment.

Values are least square means.

In the GSC group the SID Lys intake (g/d) of IPF pigs increased from the first to the second week (*P *< 0.05) and from the third to the fourth week (*P *< 0.05). Meanwhile, the SID Lys intake of GPF pigs placed in the GSC barn increased weekly (*P *< 0.05), reaching its greatest amount at the last experimental week (*P *< 0.05). As a result, GPF pigs ingested more (*P *< 0.05) SID Lys than IPF pigs during the second (28%), third (52%), and fourth (62%) experimental weeks (*P *< 0.05). Group-phase and IPF pigs had similar ADG during the first and second weeks of the trial (*P *> 0.05). However, during the third and fourth weeks, the ADG of GPF pigs was 35 and 27% greater than that of the IPF pigs, respectively (*P *< 0.05). The G:F of GPF pigs remained stable during the entire experimental period (*P *> 0.05). Conversely, the G:F of IPF pigs decreased during the third week (*P *< 0.05) and had a lower (*P *< 0.05) value compared with that of GPF pigs during the third week.

#### Poor sanitary conditions

No effects of diets or interactions between diets and feeding systems or diet and time were observed under PSC (*P *> 0.05). Interactions (*P *< 0.05) between feeding systems and time were observed for BW, body lipid, and body protein, demonstrating that the evolution of these variables over time was different between GPF and IPF pigs. Indeed, BW, body lipid, and body protein were impaired in IPF pigs (*P *< 0.05). Main effects of the feeding system were observed for PD and LD in which IPF pigs had lower (*P *< 0.05) PD and LD when compared with GPF pigs.

Under PSC, differences between feeding systems in the SID Lys intake were observed in the second and third week (*P *< 0.05). Consequently, IPF pigs ingested 25 and 32% less SID Lys than GPF pigs, respectively (*P *< 0.05). Similarly, to the GSC, the ADG of GPF and IPF pigs assigned to PSC differed in the third week (*P *< 0.05). During that week, the ADG of GPF pigs was 45% greater than that of IPF pigs (*P *< 0.05). The ADFI tended to be lower (*P *= 0.09) for IPF pigs compared with that of GPF pigs (Figure 4), but it was not affected by the AA ratios (*P *> 0.05). No differences (*P *< 0.05) were observed between FS for G:F over the weeks.

### Lysine and nitrogen balance

#### Good sanitary conditions

The IPF reduced (*P *< 0.05) the SID Lys and N intake and excretion (Table 3) in pigs raised under GSC. Specifically, pigs under GPF ingested 38% more SID Lys and 30% more CP than those fed with IPF (*P *< 0.05). Additionally, GPF pigs excreted 44% more (*P *< 0.05) N than IPF pigs. Consequently, the _k_Lys and N efficiency were greater in IPF pigs (*P *< 0.05).

**Table 3.** Lysine (Lys) and nitrogen (N) balance of pigs raised in good or poor sanitary conditions and fed with control (CN) or functional amino acid supplemented (AA+) diets in group-phase (GPF) or individual precision feeding (IPF) feeding systems^1^

	GPF		IPF			*P*-value		
Item	CN	AA+	CN	AA+	SEM	FS	Diet	FS × Diet
***Good sanitary conditions***								
** CP intake, g/d**	205.4	222.3	157.0	171.1	5.970	0.01	0.09	0.87
** N excretion, g/d**	5.72	6.39	4.23	4.16	0.223	0.01	0.27	0.36
** N efficiency, %**	55.6	54.4	57.4	60.4	0.776	0.01	0.52	0.16
** Lys intake, g/d**	13.6	14.2	9.6	10.6	0.418	0.01	0.13	0.68
**_k_Lys, %^2^**	61.9	62.7	70.9	72.9	1.148	0.01	0.42	0.74
***Poor sanitary conditions***								
** CP intake, g/d**	182.7	175.9	129.5	135.4	5.855	0.01	0.96	0.49
** N excretion, g/d**	5.34	5.00	4.33	4.28	0.162	0.01	0.52	0.62
** N efficiency, %**	51.8	54.0	45.1	48.2	1.899	0.11	0.49	0.91
** Lys intake, g/d**	12.1	11.3	8.1	8.5	0.395	0.01	0.69	0.30
**_k_Lys, %^2^**	57.8	62.5	54.0	57.7	2.200	0.35	0.36	0.91

CP = crude protein; SEM = standard error of means;

1 Data were analyzed considering feeding systems (FS), diets (Diet), and their interaction as fixed effects.

2 Standardized ileal digestible lysine utilization efficiency (_k_Lys) = (PD × 0.07)/(SID Lys intake – ((0.313 g Lys/kg DM × ADFI) + (0.0045 g Lys/kg BW^0.75^ × BW^0.75^) + (0.0239 g Lys/kg BW^0.75^ × BW^0.75^))) × 100, where PD is protein deposition, DM is dry matter, ADFI is daily feed intake, and BW^0.75^ is the metabolic body weight.

Lys and N intake and efficiency analysis were performed using only the pigs that were scanned in the DXA (10 pigs/treatment). Three pigs were removed from the nutrient balance analysis in poor sanitary condition due to lameness and arthritis: one pig from GPF_CN and two pigs from IPF_CN treatments. Additionally, two outliers were removed from the analysis in good sanitary condition: two pigs from IPF_AA+ treatment.

#### Poor sanitary conditions

Under PSC, GPF pigs ingested 41 and 35% more (*P *< 0.05) SID Lys and CP than IPF pigs, respectively. However, GSC pigs, _k_Lys and N efficiency did not improve (*P *> 0.05).

### Blood parameters

#### Good sanitary conditions

No effect of dietary treatments or interactions among feeding systems, diet, and time were observed (*P *> 0.05) for any blood parameter, regardless of the SC (Table 4). Haptoglobin and triglyceride blood concentrations in GSC pigs decreased on d 7 and remained low until the end of the trial regardless of FS and diet (*P *< 0.05). Similarly, IgA concentration reduced on d 28 (*P *< 0.05) in GSC pigs. Creatinine blood concentration increased on d 7 and remained high until d 28 (*P *< 0.05) in GSC pigs. The feeding system effect was observed for creatinine concentration, and IPF pigs had a lower creatinine blood concentration (*P *< 0.05) compared with that of GPF pigs.

**Table 4.** Blood parameters of pigs raised in either a good or poor sanitary condition and fed with a control (CN) or functional amino acid supplemented (AA+) diet in group-phase (GPF) or individual precision feeding (IPF) systems^1^

	FS		Diet		Days				*P*-value						
Item	GPF	IPF	CN	AA+	0	7	28	SEM	FS	Diet	T	FS × Diet	FS × T	Diet × T	FS × Diet × T
***Good sanitary conditions***															
** Haptoglobin, g/L**	0.43	0.41	0.39	0.45	0.49^a^	0.40^b^	0.37^b^	0.021	0.64	0.33	0.02	0.72	0.09	0.76	0.56
** Albumin, g/dL**	3.11	3.00	3.08	3.03	3.06	3.09	3.01	0.030	0.18	0.60	0.38	0.62	0.29	0.06	0.28
** IgG, g/dL**	0.89	0.94	0.91	0.93	0.90	0.89	0.96	0.020	0.35	0.70	0.21	0.58	0.71	0.31	0.87
** IgA, g/dL**	0.12	0.13	0.13	0.12	0.14^a^	0.13^a^	0.11^b^	0.003	0.33	0.63	0.01	0.78	0.09	0.71	0.93
** Urea, mg/dL**	15.4	12.9	13.5	14.8	13.8	14.8	14.0	0.564	0.08	0.38	0.62	0.37	0.11	0.67	0.31
** Creatinine, mg/dL**	1.43	1.29	1.38	1.34	1.24^b^	1.42^a^	1.42^a^	0.018	0.01	0.34	0.01	0.57	0.87	0.43	0.48
** Triglycerides, mg/dL**	39.8	43.5	41.4	41.9	53.6^a^	37.7^b^	33.7^b^	1.842	0.35	0.88	0.01	0.95	0.67	0.75	0.23
***Poor sanitary conditions***															
** Haptoglobin, g/L**	0.91	1.14	1.02	1.03	0.52^b^	1.29^a^	1.27^a^	0.065	0.10	0.93	0.01	0.27	0.38	0.92	0.43
** Albumin, g/dL**	2.96	3.02	3.00	2.98	2.97	2.98	3.01	0.038	0.51	0.87	0.92	0.13	0.89	0.71	0.76
** IgG, g/dL**	1.15	1.26	1.21	1.21	1.01 ^c^	1.19^b^	1.42^a^	0.043	0.30	1.00	0.01	0.46	0.64	0.67	0.64
** IgA, g/dL**	0.11	0.14	0.13	0.12	0.13^b^	0.15^a^	0.10 ^c^	0.004	0.01	0.70	0.01	0.50	0.09	0.58	0.95
** Urea, mg/dL**	16.5	15.8	17.0	15.3	13.6^b^	20.0^a^	15.2^b^	0.602	0.61	0.26	0.01	0.83	0.69	0.10	0.90
** Creatinine, mg/dL**	1.45	1.22	1.32	1.36	1.27^b^	1.42^a^	1.32^b^	0.017	0.01	0.24	0.01	0.31	0.68	0.71	0.25
** Triglycerides, mg/dL**	39.8	51.0	46.9	43.9	52.0^a^	46.1^a^	38.2^b^	2.431	0.06	0.60	0.04	0.99	0.20	0.23	0.55

SEM = standard error of means.

1 Data were analyzed considering feeding systems (FS), diets (Diet), time (T), and their interaction as fixed effects.

a–c Within a row, different superscripts indicate differences (*P *< 0.05) between sampling days by multiple comparison using a Tukey adjustment.

The blood parameters analysis was performed using only the pigs that were scanned in the DXA (10 pigs/treatment). Three pigs were removed in poor sanitary condition due to lameness and arthritis: one pig from GPF_CN and two pigs from IPF_CN treatments.

Values are least square means.

#### Poor sanitary conditions

In the PSC pigs, we observed a significant time effect for haptoglobin, which increased (*P *< 0.05) on d 7 and remained high (*P *< 0.05) until d 28. Regardless of FS and diet, PSC pigs exhibited a linear increase in serum IgG concentration over time (*P *< 0.05), reaching its peak on d 28. The main effects of time were also observed for IgA, urea, and creatinine regardless of FS and diets (*P *< 0.05). All three of these parameters exhibited a quadratic pattern over time with elevated values observed on d 7 (*P *< 0.05), followed by a decrease on d 28 (*P *< 0.05). On the other hand, we noticed a time effect for the triglycerides serum concentration, which decreased (*P *< 0.05) on d 28 for PSC pigs. Interestingly, IPF pigs elicited greater (*P *< 0.05) IgA serum concentration independently of the sampling day or dietary treatment. Similarly, to the GSC pigs, IPF pigs in the PSC had lower (*P *< 0.05) creatinine when compared with GPF pigs regardless of the sampling time.

## Discussion

### Response of pigs to the sanitary conditions

The sanitary challenge model used in the PSC barn aimed to replicate on-farm conditions in which pigs are infected with an oral–fecal pathogen in an environment with poor cleaning practices. Even though we did not perform statistical analyses to compare the SC, as the pigs were in two different buildings, the different patterns in the analyzed variables illustrate a difference in the health status between conditions. The success of *Salmonella* Typhimurium inoculation was confirmed by the high number of positive pigs and elevated shedding levels observed on d 3.

As expected, we observed an increase in the rectal temperature within 24 h after introducing the pigs to the PSC. Similar increases in rectal temperature were also observed by authors who used a similar sanitary challenge approach (Rodrigues et al., 2021; Valini et al., 2023). Fever is a physiological response that occurs during systemic immune activation to transform the organism in a hostile environment to pathogen proliferation (Sandberg et al., 2007). The decrease in *Salmonella* detection and return of the rectal temperature, IgA, urea, and creatinine to basal values on d 28 indicate that the immune activation was more pronounced at the beginning of the trial. However, the pigs under PSC showed an increase of 250% in haptoglobin concentration on d 7 and 246% on d 28 when compared with d 0. Haptoglobin is synthesized in the presence of pro-inflammatory cytokines and it is considered a blood indicator of health status in pigs (Johnson, 1997; Le Floc’h et al., 2009). This indicates that even with the smaller immune activation after the first week of challenge, the PSC pigs seemed to be challenged for the duration of the experimental period.

Increased IgG and IgA concentrations in PSC pigs indicates that the SC was able to induce a humoral immune response. Unlike IgG, the IgA concentration decreased on d 28. IgA is synthesized in mucosa-associated lymphoid tissues in the presence of pathogens (De Sousa-Pereira and Woof, 2019). Therefore, the decrease in the number of positive pigs and shedding of *Salmonella Typhimurium* may have reduced the stimulus to its synthesis. Moreover, the longer half-life of IgG compared to that of IgA in circulation (Schroeder and Cavacini, 2010; De Sousa-Pereira and Woof, 2019) may also explain the earlier decrease in IgA levels.

Immune system activation increases mobilization of body reserves (e.g., muscle and adipose tissues) to provide nutrients to support the immune response (Campos et al., 2017; Le Floc’h et al., 2018). However, muscular AA profile differs from the AA profile required by the immune system, inducing an AA imbalance that increases AA oxidation and urea synthesis (Reeds et al., 1994). Even though we did not directly measure muscle proteolysis, the observed changes in creatinine and urea serum concentrations over time are indicators of muscle breakdown and AA oxidation. Creatinine is formed in muscle from the spontaneous and irreversible degradation of creatine and phosphocreatine, compounds involved in cellular energy metabolism. Since its production is proportional to muscle mass, serum creatinine concentration has been commonly used as an indirect indicator of muscle proteolysis (Yin et al., 2017). The use of dietary AA and body reserves to assemble the immune response impairs PD and N retention (Barcellos et al., 2021; Valini et al., 2023). In this trial, although we have not compared SC, we noticed that PSC pigs exhibited numerically lower PD, _k_Lys, and N, efficiency compared with GSC pigs. This potentially supports the idea that dietary energy and nutrients are preferentially used by the immune system and that the unused ones can be used for growth or other productive functions.

On the other hand, despite the evidence of immune system activation, serum creatinine concentration also increased over time in pigs under GSC. Additionally, an elevated creatinine concentration was observed in GPF pigs. The GPF pigs had greater final body protein mass and PD during the trial regardless of the SC. Thus, the changes in their serum creatinine alone are likely associated with the increase in body protein mass (Remus et al., 2019a) rather than proteolysis to supply AA to the immune system.

It was previously demonstrated that an immune challenge increases the energy requirement for maintenance (Huntley et al., 2018). Similarly to the skeletal muscle, immune system activation promotes lipolysis while inhibiting lipogenesis, thereby increasing the availability of fatty acids that can be used as an energy source (Guo et al., 2015; Valini et al., 2023). Although plasma triglyceride concentration decreased on d 28 in both SC, PSC pigs showed a numerical increase on d 7 compared to GSC pigs. Considering that the highest *Salmonella* shedding and fever occurred during the first week, this transient numerical increase in triglycerides may reflect reduced lipogenesis and a greater availability of energy to support the acute immune response. Altogether, these results indicate a successful immune system activation in pigs under PSC.

### Response of pigs to different feeding systems

The mathematical model developed to estimate individual nutrient requirements by Hauschild et al. (2012) was validated and tested in different trials (Andretta et al., 2014; Cloutier et al., 2015; Andretta et al., 2016), consistently demonstrating its ability to reduce the excess of nutrients provided while sustaining growth performance (Santos et al., 2018; Remus et al., 2019a,b). Therefore, we hypothesized that IPF is an alternative feeding strategy that could be used for challenged pigs once the immune system activation increases the population variability (Van der Peet-Schwering et al., 2019; Ortiz et al., 2024).

In the present study, IPF improved the efficiency of N and Lys utilization in pigs raised under GSC. However, regardless of sanitary status, pigs fed according to the IPF strategy exhibited reduced growth performance compared with those under GPF. As ADFI did not differ between feeding systems under either condition, these results indicate that the observed differences in growth performance were primarily driven by the dietary AA profile and SID Lys concentration rather than feed intake. The reduction in growth performance observed when the Thr: Lys and Met:Lys ratios were 0.65 and 0.36, respectively, for IPF pigs in the CN group compared with GPF CN pigs was expected. Previous studies have shown that the minimum Thr: Lys ratio required to maximize ADG in healthy pigs is approximately 0.82 (Remus et al., 2019a), while the optimal Met: Lys ratio is around 0.38 (Remus et al., 2019b). Consistent with this, the BW, ADG, and G:F of IPF pigs under GSC increased when they were fed AA+ diets, confirming that the original IPF diets were deficient in key AA. The improvement in performance with the AA+ diets, despite no change in Lys intake, supports the hypothesis that AA supply, rather than energy or Lys intake, was the limiting factor in GSC. These findings are aligned with those observed by Remus et al. (2019a,b), who demonstrated that, in IPF, pigs have a linear response when fed with 30% more Thr and Met:Lys. Nonetheless, the full growth response to improved AA profiles may be constrained when lysine becomes the first limiting AA instead of the tested amino acid. This appears to be the case in the present experiment, particularly under PSC, where Lys intake was markedly reduced due to the nature of the enteric challenge and the way the IPF model responds to abrupt decreases in ADFI and ADG. Such effects are further exacerbated when the dietary AA profile required to support growth is itself limiting. Altogether, these results highlight the importance of applying distinct AA ratios in IPF systems for healthy pigs and for those exposed to suboptimal or immune-challenging conditions.

Compared with GSC, we observed a decrease in the ADFI for pigs under PSC in the first experimental week, which could be attributed to immune system activation (Johnson, 1997). However, a similar, though less pronounced, ADFI reduction was also observed in GSC pigs, which was unexpected. In the absence of immune activation markers, this reduction was likely due to the transition from adaptation to experimental diets and stress associated with intensive handling and sampling at the beginning of the trial. The differences in the ADG between GPF and IPF pigs during the trial were more pronounced under PSC (24%) than under GSC (17%). Conversely, the ADG of the IPF and GPF pigs were similar in the last week of the trial under PSC. This suggests that, apart from the ADG reduction in the first week, another factor might have influenced the performance of IPF pigs under PSC. During immune activation, there is often a reduction in the ADG due to the redirection of nutrients from growth to the immune system. The amount of nutrients required for immune responses depends on the challenge imposed (e.g., lipopolysaccharide, poor hygiene, pathogen inoculation), pathogen (e.g., virulence, concentration), and individual responses (Sandberg et al., 2007). These factors are not accounted for in the current IPF model. As previously discussed, the AA ratios used in the AA+ treatment remained below the levels recommended to maximize growth performance in healthy pigs (Remus et al., 2019a,b). However, as a proof of concept, identical AA ratios were applied in both the IPF and GPF feeding programs. Consequently, IPF pigs raised under PSC received diets that did not fully meet their requirements for growth and, even less so, accounted for the additional metabolic demands associated with immune system activation. Hence, IPF pigs may have to mobilize more body reserves to support their immune response. Indeed, IPF significantly reduced growth performance, PD, and LD in PSC pigs compared with GPF pigs. The absence of a statistical difference in ADG in the last week may have occurred due to the resolution of the immune system activation that increased nutrient availability for growth.

Moreover, to account for the variability between individuals, the IPF model forecasts a 1-d prediction of DFI and 7-d prediction of BW until new real-time data are acquired (Hauschild et al., 2012). These data are used to determine the required individual dietary SID Lys concentration. However, data collected during periods of growth disruption (e.g., health challenges, handling procedures) was also used as input to predict future growth performance. Such changes in growth directly affect the empirical model estimations for DFI and DG, and the observed discrepancy in Lys intake suggests that the model may have inaccurately estimated SID Lys requirements of IPF under both GSC and PSC. This also explains the lowered growth performance of GPF pigs in GSC. The error is exacerbated by the fact that feed intake is captured daily, while BW is captured weekly due the lack of reliable real-time weight measures. This temporal misalignment in the two primary variables hinders the accuracy of predictions. Although the recovery of DFI can be observed on a day-to-day basis, the DG, which is the main driver of SID Lys requirements in the model, will only be available 7 days later. This impairs the ability to correct SID Lys predictions in the short term when the animals recover their regular performance after a disturbance. This limitation is evidenced by the greater ADG and BW of IPF pigs that received AA+. These findings highlight the need for robust real-time data collection and the need for improvements in the empirical component of the model for practical use (Hauschild et al., 2020). It was the first time that the IPF model has been used in a drastic sanitary scenario out of optimal conditions. The model has a smoothing component used to minimize the influence of potential drastic changes of BW and ADFI on AA estimation (Hauschild et al., 2012). The results observed herein indicate that the smoothing component used in previous trials (Andretta et al., 2014; Santos et al., 2018; Remus et al., 2019a,b) is not adequate when a high magnitude of BW and ADFI fluctuations are encountered. Therefore, caution should be used when applying the current IPF model to pig populations subjected to sanitary or other challenges changing feed intake patterns or animal performance. As future perspectives, the IPF model will be updated based on the findings of this experiment to consider real-time losses of growth performance during sanitary challenges.

### Effect of functional amino acid supplementation

It has been previously shown that immune system activation modifies the AA requirements of pigs (Jayaraman et al., 2015; Jayaraman et al., 2017). Such modifications are related to the fact that pigs require AA to build an immune response (Reeds et al., 1994; Van der Peet-Schwering et al., 2019). Additionally, an extra supply of FAA as Met, Thr, and Trp are used in specific metabolic functions that allow pigs to cope with the negative effects of immune system activation (Le Floc’h et al., 2018; Rodrigues et al., 2021; Valini et al., 2023).

For instance, Met is a precursor of glutathione peroxidase, an antioxidant enzyme that neutralizes reactive oxygen species produced during the immune challenge (Le Floc’h et al., 2018). Threonine is the major component of mucin, which prevents microorganism translocation from the intestinal lumen to the bloodstream (Koo et al., 2020). Finally, Trp down-regulates T-cell proliferation and prevents an exacerbated immune response (Le Floc’h et al., 2018). In this sense, we expected that the combined supplementation of Met + Cys, Thr, and Trp above their optimal ratio for growth would alleviate the negative effects of immune system activation as observed by other authors (Van der Meer et al., 2016; Rodrigues et al., 2021; Valini et al., 2023). However, in this experiment, feeding pigs with FAA did not influence blood parameters or performance variables under PSC.

Furthermore, most of the studies which evaluated the combined effect of increasing 20% of the Met + Cys, Thr, and Trp to Lys ratio used the AA ratios recommended by the NRC (Van der Meer et al., 2016; Rodrigues et al., 2021; Valini et al., 2023). To the best of our knowledge, this is the first study to evaluate the supplementation of functional AAs using diets formulated with the suggested InraPorc AA ratios. The Met + Cys (0.60), Thr (0.65), and Trp (0.20) to Lys ratios are 7%, 8%, and 15% greater than the NRC (2012) values, respectively. Therefore, the absence of an FAA supplementation effect in this study might be related to the fact that the CN diet was already providing the FAA to support the immune response. As well, it is not discarded that the lower percentage of positive *Salmonella* Typhimurium cases in the GPF pigs fed CN diet might have masked the potential gains of the AA+ treatment.

Additionally, to create the PSC, we followed the protocol developed by Valini et al. (2023), as described above. The intensity of the immune activation influences the maintenance requirements and the rate at which AA are required for the synthesis of immune components (Pastorelli et al., 2012; Van der Peet-Schwering et al., 2019). As an example, in a meta-analysis performed by Pastorelli et al. (2012), the authors demonstrated that the changes in maintenance account for 74% of the reduction in growth performance caused by digestive bacterial infections. However, the same authors showed that this percentage is reduced to 30% in respiratory diseases. Given the complexity of the immune response, supplementing only these three amino acids may not be enough to show clear benefits, as they act on specific targets and cannot fully counter the broad metabolic changes caused by the challenge. Thus, even when FAA are supplemented, the intensity of the challenge may hide its benefits for growth performance due to its redirection for the synthesis of other proteins than those mentioned above.

Finally, the immune response is a dynamic process with feedback mechanisms that enhance activation in the presence of an antigen and decrease activation once the antigen is neutralized (Johnson, 1997). The rectal temperature, positive pigs for *Salmonella* shedding, and blood parameters observed confirm that the acute phase of the sanitary challenge occurred in the first experimental week. During this period, GPF pigs fed either AA+ or CN diets consumed similar amounts of FAA. The similarity in FAA intake between CN and AA+ pigs can be attributed to the lower ADFI of CN pigs in the first week of challenge. Pigs have different abilities to cope with sanitary challenges. Therefore, even when subjected to infections with the same pathogen, pigs exhibit different degrees of feed intake reduction (Sandberg et al., 2007; Ortiz et al., 2024). As a result, CN and AA+ pigs had similar amounts of FAA available to synthesize immune system components during the acute phase of the sanitary challenge. Such results may have prevented better immune protection in AA+ pigs, resulting in no significant difference compared with the CN group. Thus, our findings suggest that the benefits of FAA for immune-challenged pigs may depend on the AA ratios used to formulate diets, the intensity of the immune activation, or the AA intake during the challenge.

## Conclusion

In conclusion, the combined effect of precision feeding and increased FAA ratios to Lys did not alleviate the negative impacts of immune system activation in growing pigs, nor increase their nutrient efficiency. Increasing the AA ratios of Met + Cys from 0.60 to 0.72, Thr from 0.65 to 0.78, and Trp from 0.20 to 0.24 did not reduce immune activation. This could be due to the AA ratios used in the diets, the intensity of the immune response, or the potential impact on AA intake during the sanitary challenge. Large fluctuations in individual BW and DFI hinder the empirical model’s ability to correctly forecast these variables, resulting in poor estimations of the SID Lys requirement in the IPF system. This study highlights that, although complex and not fully understood, the immune system’s demand for nutrients can impose significant cost for pigs, especially when dietary nutrients are restricted. Despite the complexity, these requirements must be included in our models when they are to be used on commercial farms, particularly those that use precision feeding systems.

## Abbreviations

CFU: colony forming units
D: diet
DM: dry matter
ADFI: average daily feed intake
ADG: average daily gain
FAA: functional amino acids
GPF: group phase feeding
FS: feeding system
GSC: good sanitary condition
IPF: individual precision feeding
LD: lipid deposition
N: nitrogen
Nal+: nalidixic acid
NE: net energy
PBS: phosphate buffer saline
PD: protein deposition
PSC: poor sanitary condition
SC: sanitary condition
STTD: standard total tract digestible
